# Interfacial Structures and Properties of Organic Materials for Biosensors: An Overview

**DOI:** 10.3390/s121115036

**Published:** 2012-11-06

**Authors:** Yan Zhou, Cheng-Wei Chiu, Hong Liang

**Affiliations:** 1 Materials Science and Engineering, Texas A&M University, College Station, TX 77843, USA; E-Mail: yanzhou@tamu.edu; 2 Department of Mechanical Engineering, Texas A&M University, College Station, TX 77843, USA; E-Mail: helloweiwei6824@tamu.edu

**Keywords:** biosensor, interfacial properties, organic materials

## Abstract

The capabilities of biosensors for bio-environmental monitoring have profound influences on medical, pharmaceutical, and environmental applications. This paper provides an overview on the background and applications of the state-of-the-art biosensors. Different types of biosensors are summarized and sensing mechanisms are discussed. A review of organic materials used in biosensors is given. Specifically, this review focuses on self-assembled monolayers (SAM) due to their high sensitivity and high versatility. The kinetics, chemistry, and the immobilization strategies of biomolecules are discussed. Other representative organic materials, such as graphene, carbon nanotubes (CNTs), and conductive polymers are also introduced in this review.

## 1. Introduction

**Figure 1.**
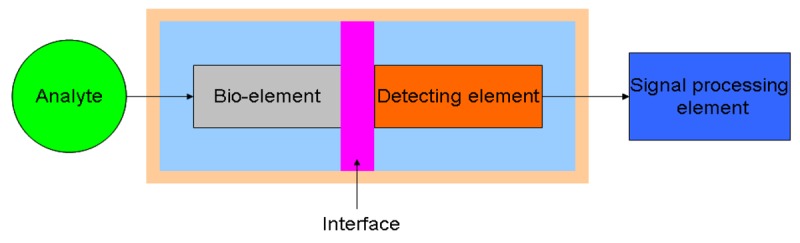
Schematic representation of the basic concept of a biosensor (adapted from [18]).

A biosensor is a device for transforming biological signals into analytical ones. It combines a bio-component with a physical element that is mainly used for converting the complex biologically derived message to quantitative information. A biosensing device has a wide range of application in the fields of environmental monitoring, drug development, and biomolecular interactions. The representation of a biosensor consists of three major components: a sensitive bio-element, a detecting element, and a signal processing element. The bio-element can be enzymes, living cells, or microorganisms, *etc.* [1–3], which recognizes the target analyte. The detecting element can be used to monitor the variation of electric current and potential [4–7], impedance [8–11], optical intensity [12–15], and electromagnetic radiation [16,17], among others. The bio-element directly interfaces to a signal transducer (detecting element), which together relate the variation of the analyte to a measurable response. Different constitutions of a bio-element coupled to a detecting element lead to a variety of applications. Figure 1 is the schematic diagram that represents the concept of a biosensor.

## 2. Types of Biosensors

Due to the different signal detecting mechanisms, biosensors can be categorized into various types, including resonant, photometric, thermal detection, ion-sensitive field-effect transistors (ISFETs), and electrochemical sensors. In the following sections, the types used for biosensors and their sensing mechanisms will be discussed.

### 2.1. Resonant Biosensors

A resonant biosensor is generally used to detect bio-relevant molecules in aqueous media. The sensing focuses on the change of mass, viscosity, or conductivity of the substrate surface. Many biosensors fall into this category, which includes surface acoustic wave sensors, magnetoelastic sensors, quartz crystal (piezoelectric) biosensors, *etc*.

**Figure 2.**
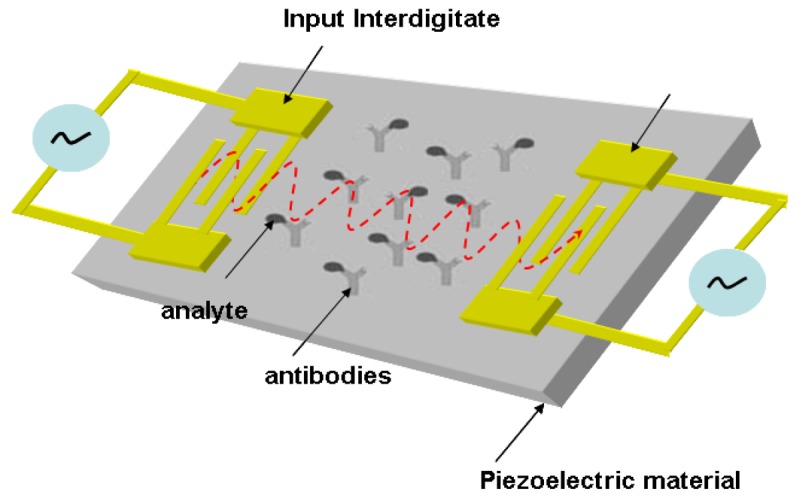
Schematic representation of a SAW-based biosensor (adapted from [26]).

The prototype of a surface acoustic wave sensor (SAW) was established based on a strongly confined acoustic energy detected by interdigitated transducers [4,19,20]. A SAW sensor has one piezoelectric material positioned in between two transducers. An input transducer electrically excites acoustic wave of the piezoelectric material, in which the acoustic wave is received at the output transducer. The wave energy radiates into the aqueous bulk due to the perpendicular displacement of the wave propagation in aqueous environment. This causes a high attenuation of the received signal, which hinders the application transferred into a biosensor [4,21,22]. The shear-horizontal (SH) surface wave, generally referred to as a Love wave, is generated using a deposited elastic layer to guide the direction of the acoustic resonance. The elastic material significantly reduces the spreading loss of acoustic energy [23–25]. Now the operation of a SAW-based biosensor is driven by the coupled wave transducer and antibody on a piezoelectric substrate. The antibody used as a bio-element is immobilized on the device that catches analytes from the aqueous medium. The bonded analytes will change the velocity of the SAW, which alters the output signal generated by the integrated electronics. The variation of the output signal can be used to evaluate the concentration of the analytes. The schematic setup of a SAW-based biosensor is presented in Figure 2 [26].

The theoretical principal of quartz crystal biosensors relies on the piezoelectric effect of the crystal. When a piezoelectric material is subjected to an AC potential, a mechanical oscillation of the material is excited. The frequency change of such oscillation is correlated to the mass change on the material surface. The mathematical relationship (Equation (1)) between the resonant frequency and the mass variation was firstly described by Sauerbrey [27]:

(1) $$\Delta f_{m}=\frac{{f_{0}}^{2}}{F_{q}\rho_{q}}m_{s}$$

where *f_0_* is the resonance frequency of the quartz resonator, F_q_ is the constant of crystal frequency, ρ_q_ is the material density, and m_s_ is the mass/area. In the quartz biosensor, the adsorption of target analytes onto the quartz surface causes the shift of resonant frequency that can derive information for quantitative analysis.

The operation of magnetoelastic sensors relies on the mechanical vibration of a magnetoelastic material when the material is subjected to a magnetic field. Such responses of the sensor not only can be detected acoustically, but also magnetically. The vibration generates an elastic wave within the magnetoelastic material that results in a detectable magnetic flux [28]. In a magnetoelastic sensor, the vibrational frequency (*f*) can be expressed as[29]:

(2) $$f=\frac{1}{2L}\sqrt{\frac{E}{\rho }}$$

where L is the length, E is elasticity, and ρ is the density. When a magnetoelastic sensor is subjected to a mass change (Δm), the variation of frequency can be expressed as[29]:

(3) $$\Delta f=-f_{0}\frac{\Delta m}{2m_{0}}$$

where f_0_ is the initial resonance frequency. When the sensor is subjected to a damping effect created by the surrounding medium, the shift in resonance frequency can be expressed as [30]:

(4) $$\Delta f=\frac{\sqrt{\pi f_{0}}}{2\pi \rho_{s}d}\sqrt{\eta \rho_{1}}$$

where η is viscosity of medium, s_l_ is the density of medium, ρ_s_ is the density of sensor material, and d is the thickness of the sensor. At present, the characteristics of magnetoelastic materials have been employed in different kinds of bio-sensing such as glucose, avidin, *Escherichia coli*, and platelet-rich plasma [30–33], among others.

### 2.2. Thermal Detection Biosensors

Surprisingly, even with their poor reputation for weak sensitivity and non-specific heating effects, the applications of thermal biosensors still draws considerable attention. A thermal biosensor is a promising analytical tool due to the following advantages:

- No chemical contact between transducer and sample leading to long-term stability.
- Economical bulk products and quick response.
- Measurements are not interfered by sample characteristics.

Novel thermal biosensors based on enzymatic conversion have been developed for monitoring enzyme reactions. This is mainly because most biochemical reactions have an exothermic character. The principle of thermal biosensor measurement is based on the first law of thermodynamics:

(5) $$\text{Q}=-{\text{n}}_{\text{p}}\sum \Delta \text{H}$$

(6) Q=CpΔT

where Q is the total energy of heat, H is the enthalpy, Cp is the heat capacity. The measureable local temperature shift (ΔT) generated by the heat production is dependent on the heat capacity of the system [34]:

(7) $$\Delta \text{T}=-\Delta {\text{Hn}}_{\text{p}}/\text{Cp}$$

**Figure 3.**
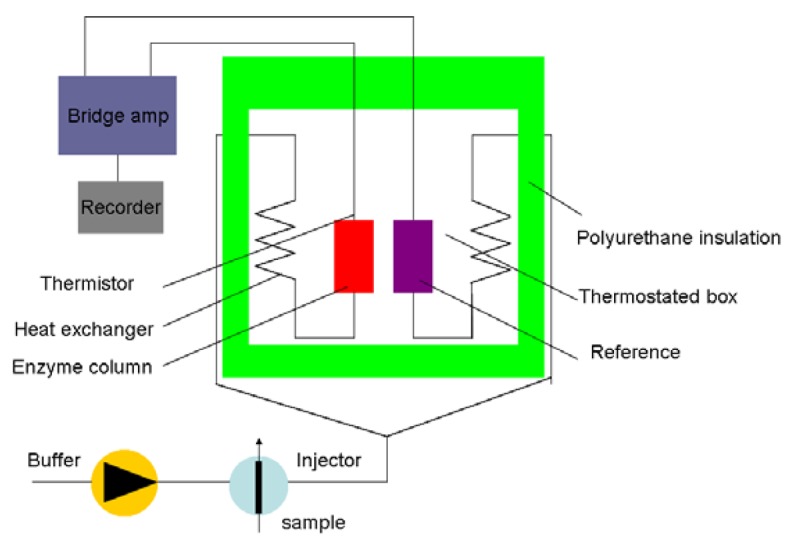
Schematic representation of a thermal biosensor (adapted from [36]).

Generally, the Cp of most organic media is lower than that of the aqueous solvent [35]. The sensitivity and detection limit of the sensor are determined by the organic solvents. Figure 3 shows a schematic diagram of the principle set-up for an enzyme thermistor (ET). The thermostated box controls the physiological temperature. Samples and the buffer are injected in the ET where the aluminum thermostates the buffer stream. The heat generated by the enzymatic conversion reduces thermistor resistance and the bridge amplifier registers the signal.

### 2.3. Photometric Biosensors

**Figure 4.**
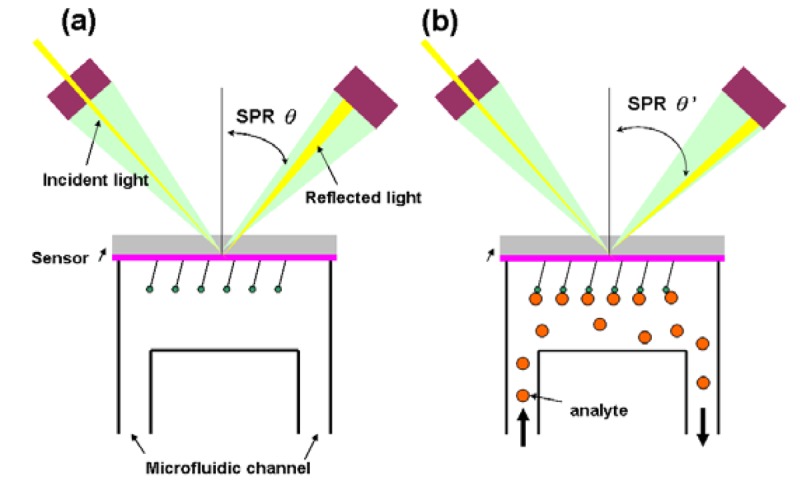
Schematic representation of a SPR biosensor (adapted from [42]).

In this type of biosensor, the measured output transduced signal is the light intensity. More than 75% of the research papers for optical biosensors focus on using the surface plasmon resonance (SPR) [37] effect. The SPR essentially is a diffraction anomaly due to the surface excited plasma [38]. The electrons resonate when the wavelength of oscillating mobile electrons (plasma) matches the wave vector of incident light. The resonating plasma is associated with the electromagnetic waves propagating in a direction parallel to the interface of two media, and decaying evanescently, *i.e.*, evanescent wave. Due to the limited propagation length of a surface plasma wave (SPW), the detection of SPR sensor is conducted where the SPW is excited by the incident light source. A SPR sensor is constituted by an incident light, a transducer with a gold side contacted with the detection apparatus and the other side contacted with microfluidic system (flow side), and an electronic system for processing the output signal. Fixed wavelength is shot to the gold side and is reflected, which induces an evanescent wave penetrating into the flow side. During the measurement, the analyte is introduced through the microfluidic channel and bound with the sensor, which changes the dielectric constant of the medium. This will lead to the changes of refractive index near the surface hence affecting the refracting SPR angle (Figure 4) [39–41].

The sensing mechanims of other photometric biosensors are based on the optical properties of analytes, which include absorbance/scattering and fluorescence. The utilization of the absorbance properties of analytes is to detect the amount of laser light being blocked or transmitted by the target cells [43,44]. Such a measuring system provides a rapid, simple, repeatable, and label-free assay for the immobilized analytes. Due to the limited sensitivity of the conventional methods in absorbance studies, an optical waveguide has been introduced in order to enhance the efficiency of absorbance biosensor. Optic fiber probes with different geometries have been employeed in absorbance biosensors that include coiled and tapered fibers, among others [45,46]. The fluorescent staining technique is also introduced in photometric biosensors. Such a technique is usually requred in microfludic-platform biosensors for the purpose of bacterial counting [47].

### 2.4. Ion-Sensitive FETs Biosensors

The development of ISFETs, usually used for pH and ion concentration measurements [48–50], started in the 1970s. To integrate the sensing circuit, most ISFETs were produced through MEMS fabrication based on silicon substrates. This also makes ISFETs a perfect transducing element for biosensing. The reactive silanol (SiOH) groups on the SiO_2_ surface provide a stem for covalent attachment of bio-molecules by using H^+^ and OH^+^ as binding sites:

$$\begin{array}{l} \text{SiOH}‐‐‐‐‐‐‐‐‐‐‐{\text{SiO}}^{-}+{\text{H}}^{+}\\ \text{SiOH}+{\text{H}}^{+}{‐‐‐‐‐‐‐‐‐\text{SiOH}}^{2+} \end{array}$$

**Figure 5.**
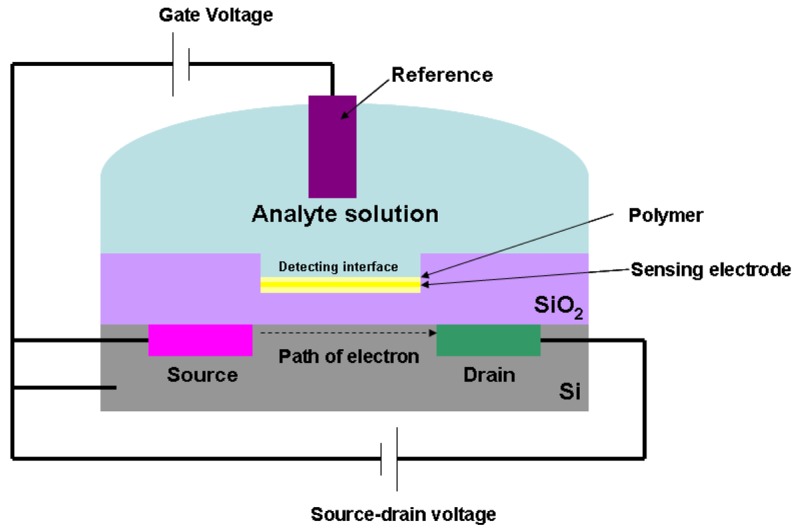
Schematic configuration of an ISFETs biosensor (adapted from [51]).

Through silicon surface modification, the analytes can be successfully immobilized onto the gate surface [51–55]. Figure 5 outlines the schematic configuration of ISFETs.

An ISFET consists of a sensing electrode coated with a polymer selectively permeable to analyte ions, and a field effect transistor structure. The current flow in the gate voltage is regulated by the potential difference between source and drain. When the analyte ions diffuse through the ion-sensitive polymer, the charged biomolecules cause the depletion or accumulation of charge carriers. This will lead to the potential difference at the detecting interface. This interfacial potential difference is regulated by the ions concentration in the solution; therefore, the change of the current in the transistor is a measure of analyte concentration.

### 2.5. Electrochemical Biosensors

**Figure 6.**
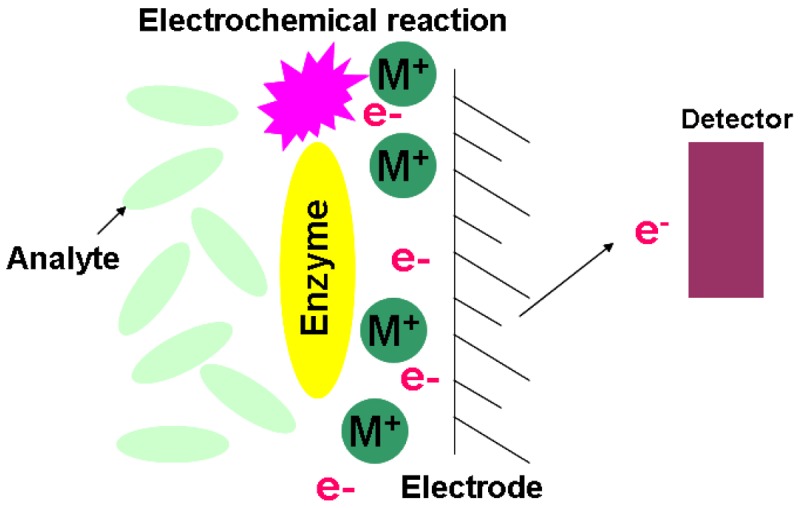
Schematic configuration of an electrochemical biosensor.

The constitutional concept of an electrochemical biosensor is based on a matrix bound bioactive material coupled with an electrochemical transducer. Essentially, it is a surface modified electrical conductor for different electrochemical functions. This type of sensor targets those biological reactions that derive ionic production and consumption. This will cause the charge transfer across the double layer of the physio-chemical transducer that generates the measureable signal [56–58]. Based on the measured signal characterizations, the electrochemical biosensor has three main classifications of silicon based chips. Figure 6 is the illustration of the electrochemical biosensor.

#### 2.5.1. Potentiometric

The principle of the potentiometric biosensor is based on an ISFET. The output signal is generated by the potential differences of oxidation/reduction reactions. The electrochemical reaction generated ions are accumulated at the ion-sensitive membrane of the ISFET interface. When this potential is applied to the electrode, it modulates the current flow through the FET leading to a measurable potential of the detector.

#### 2.5.2. Amperometric

The high sensitivity of an amperometric sensor provides the sensing ability to detect electroactive substances in biological samples. By applying a constant potential between the sensing and auxiliary electrode, the conversion of electroactive species takes place at the electrode. This will result in electron transfer, and the current is directly correlated to the bulk concentration of tested electroactive species [56,57].

#### 2.5.3. Impedimetric

The chemical reactions resulting in either ion production or consumption will change the conductivity of the solution. The measure of solution's impedance (Z) change is introduced for this type of sensor. The sensitivity of an impedimetric sensor is relatively low since the measure of conductance is essentially non-specific. This drawback can be overcome by targeting the specific defined geometry of enzymatic reactions in a microelectronic cell [56].

The types of biosensors, sensing mechanisms, and the applications are summarized in Table 1. Beside discussed above, hybrid sensors have emerged in recent years [59–62]. These sensors contain types and mechanisms that are expected to grow in the near future.

## 3. Self-Assembled Monolayer

This section focuses on the organic materials used in biosensors. Emphasis will be given to the self-assembled monolayer (SAM), which provides molecular level control over the density and position of assembled molecules. SAM is capable of packing different types of molecules in an orderly fashion at the molecular level, which generates a multifunctional surface for multitasks. SAM is advantageous due to its simplicity of preparation, high sensitivity, and few limitations in the detection range of an analyte, and most importantly, the versatility of modification that no other organic materials could match. The assembling kinetics, the chemistry of SAM, and the immobilization strategy of biomolecules onto SAM will be discussed.

### 3.1. Introduction

By definition, SAM is a single layer of biological and/or chemical molecules formed through self-assembly. It is a part of molecular nanotechnology that attracts interest due to its simple production and versatility in molecular and reaction selection. The first study on SAM was reported in 1946 by Bigelow *et al.* who used a metal surface to absorb surfactant molecules and made a layer with monomolecules [63].

### 3.2. Structure and Assembling Kinetics

**Figure 7.**
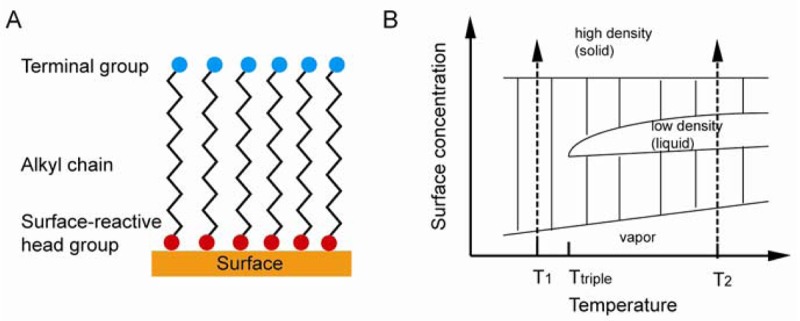
(**A**) Idealized SAM system (adapted from [64]), (**B**) Equilibrium phase diagram (adapted from [65]).

Under suitable conditions, SAMs can be directly formed on a sensor substrate. A substrate is placed in a solution containing assembling molecules, where SAM forms spontaneously. In some cases, UV irradiation, heat, voltage, and other conditions need to be applied in order to complete SAM formation. Self-assembling molecules contain three parts: the head group, which is open for bond formation with surface substrate; the alkyl chain, which is important in stabilization and order, interacts with neighboring chains through van der Waals and electrostatic forces; the functional terminal, which is open for functionalization and is used to determine the chemistry properties of SAM surface with the outer environment [64]. The idealized SAM system is shown in Figure 7(A).

As shown in Figure 7(B), there are four phases during the SAM formation, including vapor phase, intermediate phase, low density phase, and high density phase. In the vapor phase, the surface is randomly deposited with isolated molecules. In the intermediate phase, the surface is deposited by adsorbate molecules with disordered conformation. In the low density (liquid) phase, adsorbate molecules are lying down on the surface. In the high density (solid) phase, molecules are standing in order and packed with tilting angle less than 30°. SAM formation would first go through the vapor phase when the temperature is lower than the triple point, followed by the intermediate phase, and the high density solid phase last. Otherwise, the formation would incorporate a low density liquid phase if the temperature is higher than the triple point. Both processes involve the formation of solid phase islands surrounded by isolated vapor phase molecules and the nucleation and growth of these islands till the entire surface is covered. The Langmuir model Equation (8)) shown below is used to explain SAM formation:

(8) $$\frac{d\theta }{dt}=k(1-\theta )$$

where *θ* is the proportional coverage of the surface, and k is the rate constant [65]. The equation indicates that the SAM formation rate is proportional to the uncovered surface. The initial formation of alkanethiol SAMs is 80–90% complete during the first several minutes, and molecules reorganize themselves over 12–16 hours [66]; however, changes may still happen weeks later [67].

### 3.3. Chemistry of SAM

Increasing numbers of SAM systems are presented due to the accumulating knowledge in chemistry. SAM systems are separated into four types based on their chemical characteristics (Table 2). They are alkanethiol and organosulfur on a metal surface, organosilane on a hydroxylated oxide surface, alkyne and alkene on a hydride-silicon surface, and aryl diazomium salts on a carbon, metal, metal oxide, silicon surface, *etc*. In the following sections, they will be discussed in detail.

#### 3.3.1. Alkanethiol and Organosulfur

In the early days, the work of SAM was focused on the formation of organosulfur SAM on planar gold and silver surfaces through solution or vapor deposition [67]. Metal surface and assembling molecules had to be transformed into a reactive substrate to allow the formation of SAM. An increasing numbers of compound ligands and metal substrates can be used in SAM formation. However, limitations still exist in the type of ligands that can be matched to the metal at certain oxidation states. Gold is the most studied substrate due to several reasons: gold is an inert element; gold thin film is convenient to obtain and is a standard material for several characterization methods; gold shows low toxicity to biology systems and has high biocompatibility. It is also straightforward to pattern gold through lithography and chemical etching [67]. Thiol groups form gold-thiol bonds on gold surfaces with gold-thiol energies as low as 170 kJ/mol [68]. It is believed that the van der Waals force plays an important role in stabilizing SAM structures. Other commonly used and studied metals are silver, copper [69], and palladium [70]. The alkane chains in the SAM adopt trans-conformation and tilt ∼30° from normal on gold surfaces, ∼10° on silver surfaces, 12° on copper surfaces [69], and 0° on mercury [71]. Sulfonates and sulfinates, oxidized metal-thiol bonds, are formed when SAM is exposed to air. The weaker bonds between oxidized species and metal substrate lead to molecules falling from the gold surface [72]. It is shown that the length of an alkane chain is a factor in determining the rate of oxidation of a thiol group in SAM. The shorter the chain, the easier the oxidation occurs [73].

#### 3.3.2. Organosilane Based Layers

**Figure 8.**
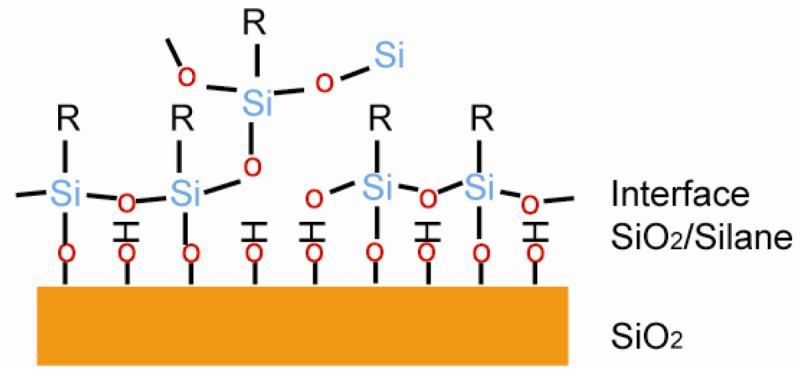
Structure of organosilane based layer. Organosilane SAM is connected to hydroxylated silicon dioxide surface through S–O bond. Some organosilane molecules were connected to the neighboring molecules (adapted from [81]).

Organosilane based monolayers are one of the most studied SAM systems. The substrate has to be a hydroxylated oxide surface, which includes silicon dioxide and other metal oxides [64]. A typical structure is shown in Figure 8, where organosilane SAM is connected to the hydroxylated surface through the S–O bond. Sagiv reported the octadecyltrichlorosilane (OTS) SAM on a hydroxylated surface. The SAM formed through a condensation reaction between the hydrolyzed OTS and the hydroxylated surface[74]. The silicon dioxide has to go through a hydrophilic treatment before usage, otherwise the uniformity of SAM would drop dramatically [75]. Less than 20% of the molecules formed S–O bonds on the hydroxylated surface, and the rest were connected to the neighboring molecules to form SAM [81].

#### 3.3.3. Hydrosilylation

**Figure 9.**
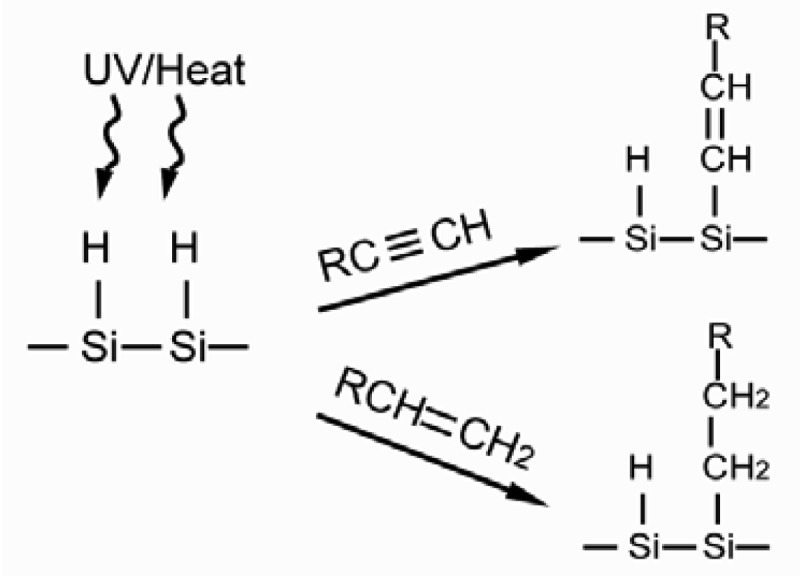
Alkyl chains of 1-alkyne and 1-alkene terminals are connected to the S-H radicals on the silicone surface (adapted from [82]).

In the preparation of SAM through the hydrosilylation reaction, the silicon surface is pretreated with UV or heat to generate the S–H radicals in order for the surface to react with alkyl chains presenting 1-alkyne and 1-alkene terminals, as seen in Figure 9. When the reaction is completed, the silicon surface is linked with alkyl chains by S–C bond and generates alkene and alkane accordingly [82]. SAM prepared by this method does not show the multilayer defect, but it has superior stability due to the non-polar bond of S–C. However, the silicon oxide largely affects the formation of the S-C bond hence reducing the quality of SAM. Thus, the SAM preparation has to be performed using oxide free silicon in an atmosphere with no oxygen [78].

#### 3.3.4. Aryl Diazonium

**Figure 10.**
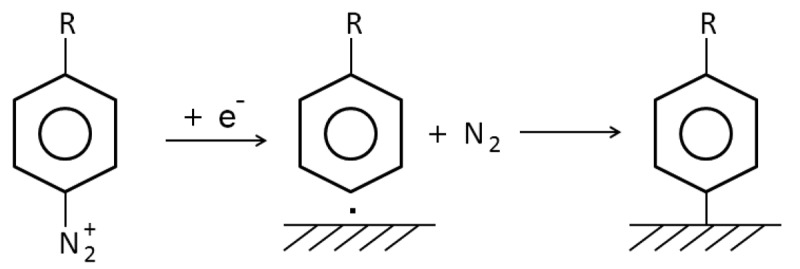
The reaction mechanism for aryl diazonium reaction based SAM (adapted from [83]).

Pinson first reported a SAM based on the aryl diazonium reaction in 1992 [79]. It involves the reduction of aryl diazonium (Figure 10), which functionalizes the carbon surface with an aromatic group, which is then open to classical chemistry reactions. This method is of interest due to SAM's capability of being applied to all carbon, silicon, metals, and metal oxides substrates. In this mechanism, it is believed that an aryl radical forms an aryl diazonium species with the release of N_2_, then a covalent bond forms between the aryl group and the substrate [80]. The resultant SAM shows higher stability, however, control over the reaction is limited.

### 3.4. Attachment of Biomolecules to SAM Biosensor Systems

Biomolecules can be attached to the functional terminals of modified electrodes by covalent and non-covalent bonds, as summarized in Table 3. Non-covalent bonds, which includes hydrogen bonds and electrostatic interactions, are widely applied in attachment of biomolecules. The attachment is relatively weak compared to a covalent bond. Nevertheless, it only needs simple reaction steps and usually is reagentless. Covalent bonds provide stronger immobilization, but are restricted to certain reactions.

#### 3.4.1. Immobilization of Biomolecules to SAM Biosensor Systems by Non-Covalent Bond

**Figure 11.**
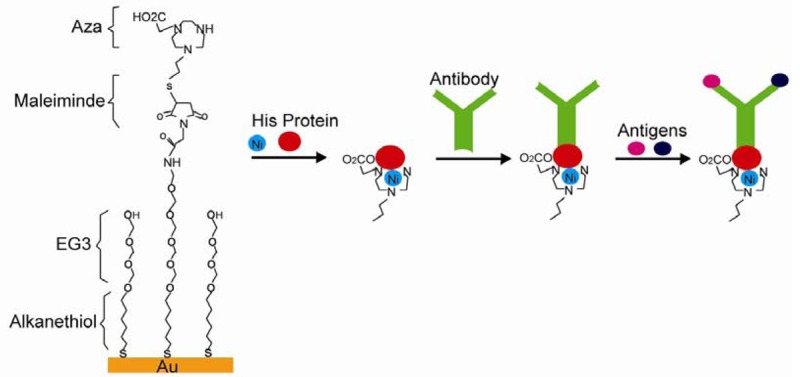
The application of non-covalent bonding in a SAM immunoassay (adapted from [89]).

Electrostatic, hydrogen, and chelation interactions are non-covalent bonds between SAM and immobilized biomolecules. Cytochrome was successfully immobilized on carboxylic terminals of alkanethiol-gold SAM through electrostatic interactions. The immobilization reaches maximum at pH values of 3.5∼5.5, where cytochrome is positively charged and attracted by negatively charged carboxylic terminals [84]. More than one species of biomolecules were immobilized through electrostatic interactions. Carboxylic terminated SAM was designed to attract lipid-DNA complexes (LDc), which showed positive charges due to excessive cationic lipids. Anionic plasmid DNA was then absorbed after the SAMs surface charge was reversed to cationic after LDc absorption, and the resulting SAM succeeded in gene transfer [85]. Hydrogen bonds were used in biomolecules immobilization, which was reported by Gomes and others. They showed a SAM system with glycan-modified surfaces and immobilized proteins through hydrogen bonds [87]. Histidine (His) modified proteins were attached to the surface through chelation interactions. For instance, nitrilotriacetic acid (NTA) was used to pretreat the quartz surface. Divalent metals such as Cu, Ni were then applied to fill tetradentate chelator sites, which formed hexagonal complexes and left two unoccupied sites for His bonding. Proteins with His tags would then be immobilized [88]. In another system showed in Figure 11, tri(ethylene glycol) and maleimide modified alkanethiol molecules were used to form SAM on a Au surface. The SAM surface was then decorated with triazacyclononane (aza) or NTA ligands. His tagged proteins were then immobilized on SAM in the presence of divalent metals followed by IgG antibodies anchored on His tagged proteins. An immunoassay was carried out based on this system, which proved His tagged proteins, IgG antibodies, and antibody specific antigens were present [89]. This type of reaction can be reversed after adding EDTA, which makes it ideal for biosensor applications [101].

#### 3.4.2. Immobilization of Biomolecules to SAM Biosensor Systems by Covalent Bonds

**Figure 12.**
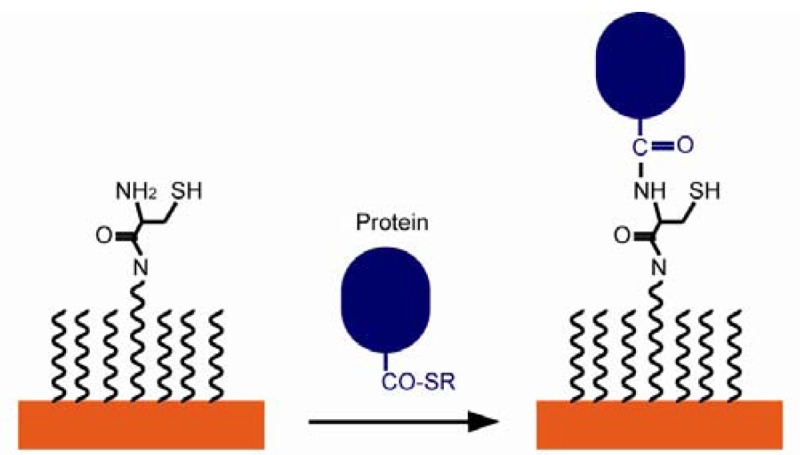
The Cys N-termini of SAM's reacts with the C-termini of protein through dehydration and forms an amide bond (adapted from [102]).

When non-covalent bonds are used in immobilizing biomolecules on biosensor surfaces, the orientations of biomolecules tend to be random. Stronger covalent bonds provide more control over the orientation. Dehydration synthesis between carboxyl groups and amino groups helps protein array to achieve certain orientation. It is a widely used strategy due to its biocompatibility, simplicity of operation, and easy access to carbodiimide agents [64]. Amino-modified SAM would react with C-termini of proteins and *vice versa*. For example, poly(ethylene glycol) (PEG) thiol SAM has Cys N-termini, which reacts with the C-termini of proteins through dehydration and forms amide bonds. The resulting immobilized proteins showed specific orientation, as seen in Figure 12 [102]. In another study, Herrwerth designed a PEG-alkanethiol SAM with carboxy termini in order to covalently couple to IgG antibodies upon chemical activation [90].

**Figure 13.**
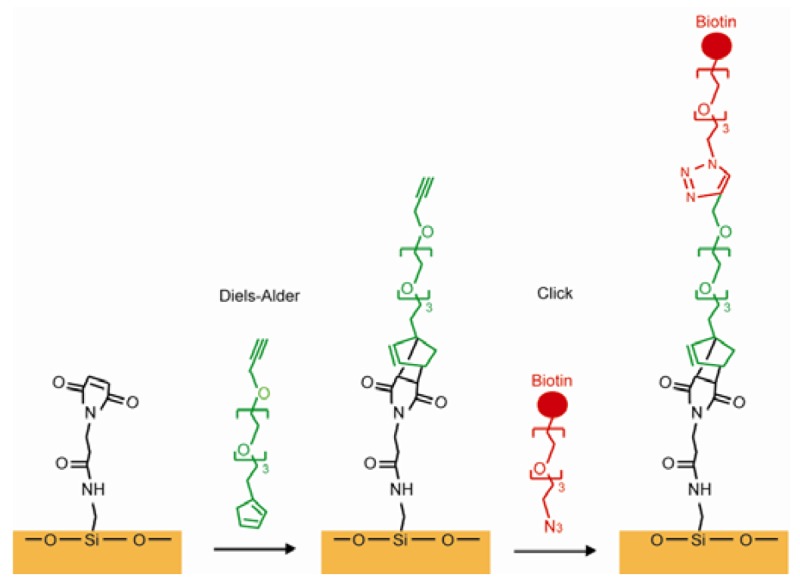
An example of the combination between the Diels-Alder reaction and the “click” reaction (adapted from [103]).

Using the reaction between maleimide and thiol residues of Cys is another common way in covalently immobilizing proteins. A hydroxylated glass surface was connected to the amino-terminated silane, and the amino group was subsequently linked to the N-succinimidyl-6-maleimidocaproate (EMCS). The thioether was produced to bind the maleimide group of EMCS on the surface and the thiol group on the Cys in the protein [92]. The “click” reaction forms covalent immobilization, which exists between alkyne and azide through cycloaddition using Cu(I) as a catalyst. This method leads to high yield with little unwanted byproducts because azido and ethynyl are rare in nature and cannot be presented as contaminations. A SAM with alkyne termini was linked with an azido modified peptide through the “click” reaction and the resulting SAM was used for a cell adhesion study [93]. Azide-terminated carbohydrates can be used to connect SAMs presenting terminal alkynes through the “click” reaction [94,95]. The Diels-Alder reaction is used to form a cyclohexene between a diene and a dieneophile. Houseman reported a process using the Diels-Alder reaction to connect a saccharide-cyclopentadiene to a benzoquinone group on the SAM. A carbohydrate array was successfully made and were capable of identifying specific lectins [97,98]. Chaikof combined the Diels-Alder reaction and the “click” reaction. A glass surface was functionalized with N-(e-maleimidocaproyl) (EMC). A PEG linker was synthesized that had a cyclopentadiene on one side and an alkyne on the other side. The cyclopentadiene was used to connect EMC by the Diels-Alder reaction and the alkyne end was designed to link the peptide or the carbohydrate by their functionalized azide group [103], as shown in Figure 13. In another synthesis route, an aldehyde-terminated alkanethiol SAM on a gold surface was used to immobilize proteins through its amine groups. The imine product was reduced to a secondary amine due to the instability of the imine group in the air [99]. This amine-aldehyde reaction was widely used in protein microarray fabrications [100]. An amine-modified single strand DNA (ssDNA) was immobilized on a SAM having an epoxy surface through a coupling reaction. However, proteins would be denatured because of the high ionic requirement of the coupling reaction [104]. Alkyne coupling was utilized as a synthesis method for connecting molecules to a SAM, which is presented by Bedyzk. An idiophenyl acetylene functionalized surface was designed to link with bromophenyl acetylene by connecting two phenyl groups with an acetylene bond [105].

**Figure 14.**
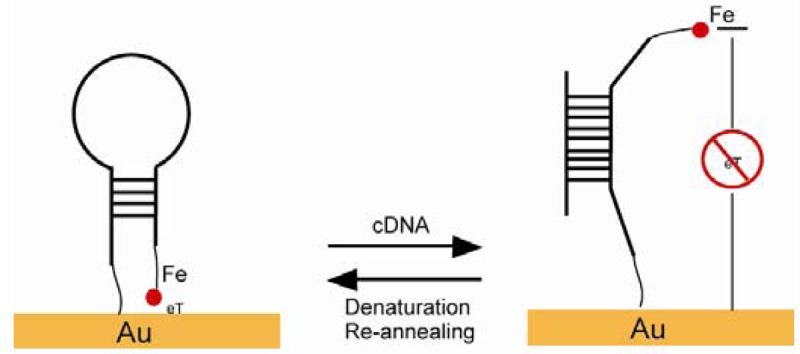
A stem-loop structure with a terminal ferrocene tag. The hybridization of target DNA changed the stem-loop structure and the electron transfer stopped, hence, the corresponding cyclic voltammogram changed (adapted from [106]).

A maleimide-derived glass surface was functionalized with EMC. The Diels-Alder reaction and the “click” reaction happened sequentially and ended with biotin termini [103]. Fan constructed a stem-loop oligonucleotide with one terminal thiol and one terminal ferrocene (Figure 14). Once the oligonucleotide self-assembled onto a gold electrode surface through a thiol group, the stem-loop structure held the ferrocene at proximity to the gold, which makes the electron transfer easy between the ferrocene and the gold. Upon target nucleotide sequence hybridization, the stem-loop structure was disrupted and a rod-like rigid structure was formed, which separated the ferrocene away from the gold electrode and caused the electron transfer abolished [106]. A similar SAM was developed with thiolated ssDNA mixed with alkanethiols as a diluent. ssDNA lied across the surface of the SAMs and prevented ions from reaching the electrode. ssDNA turned into a rod-like rigid structure upon targeting sequence hybridization and freed the space for ions to reach the electrode [107]. An excellent example of a pH sensor was presented by Wrighton and co-workers. They produced a SAM by using pH insensitive ferrocene and pH sensitive quinone. The system showed a linear response to pH changes monitored by voltammetry, where ferrocene as a reference and quinone as an indicator in addition to a large surface area as a counter electrode [108].

## 4. Other Materials

Other widely used organic biosensing materials are: graphene, carbon nanotubes (CNTs), and electrogenerated polymers. The interfaces between the organic materials of biosensors and the corresponding inorganic electrodes are highlighted.

### 4.1. Graphene

**Figure 15.**
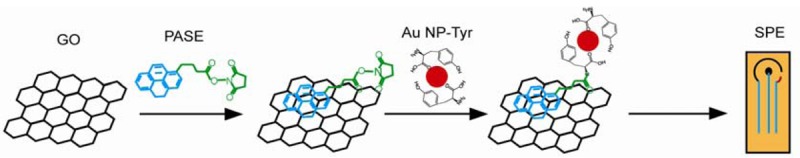
Modified GO was coated on SPEs (adapted from [114]).

Graphene was first discovered by Novoselov in 2004 [109]. The superior electron mobility, biocompatibility, and flexibility of graphene qualify it as an ideal material for biosensors. In a glucose biosensor application, graphene was modified by polyvinylpyrrolidone (PVP) and added to a certain ionic liquid (IL). The solution of PVP-graphene-IL was dropped on a glass carbon electrode (GCE) and was allowed to dry before detecting glucose. A linear response for glucose detection from 2 mM to 14 mM was recorded [110]. In a similar report, a graphene-chitosan solution was dropped onto a GCE and allowed to dry. Glucose oxidase was then coated on the graphene-chitosan-GCE. The glucose detection range was from 0.08 mM up to 12 mM [111]. Zeng functionalized graphene with sodium dodecylbenzenesulphonate (SDBS). Horseradish peroxidase (HRP) and SDBS-graphene self-assembled on the surface of a GCE. The resulting sensor showed high sensitivity and a H_2_O_2_ linear response [112]. Another graphene-based biosensor for H_2_O_2_ detection incorporated metallic nanoparticles. The solution, which contained graphene, HRP, and chitosan, was casted on a GCE. Au was electrodeposited on the surface of the modified GCE and clusters of Au nanoparticles were later formed. The H_2_O_2_ detection range was from 0.005 mM to 5.13 mM with a detection limit as low as 1.7 μM [113]. A screen printed electrode (SPE) was used as a disposable sensor system shown by Song (Figure 15). 1-Pyrenebutanoic acid succinimidyl ester (PASE) has a pyrenyl group, which interacted strongly with graphene, and a succinimidyl ester group, which reacted highly with the amines substitution. Tyrosinase functionalized Au NPs were mixed with PASE modified graphene oxide (GO). The solution of the mixture was dropped on the working SPE for catechol monitoring. The resulting sensor showed high stability and sensitivity [114].

Some graphene biosensors are electrodeless. ssDNA molecules were connected to certain dyes. GO bonded to the ssDNA labeled dye and quenched the dye's fluorescence. When the targeted ssDNA hybridized to the ssDNA-dye-GO complex, the double stranded DNA-dye was released from the GO and the fluorescence was enhanced [115]. In a biological environment, it is difficult to differentiate dopamine from its coexisting ascorbic acid (AA) and uric acid (UA) because of the overlapping voltammetric responses. A graphene nanoflake film was synthesized on a Si substrate by microwave plasma enhanced CVD. The film was shown to be capable of determining dopamine in the presence of AA and UA with high sensitivity [116].

### 4.2. Carbon Nanotube

**Figure 16.**
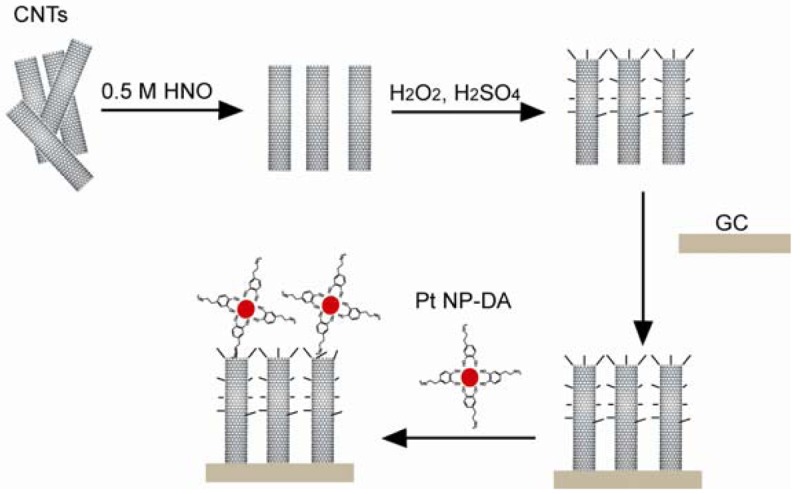
The schematic of the fabrication of a CNT based H_2_O_2_ sensor, (A-C) CNTs were separated, cleaned, and attached to the surface of a GC; dopamine (DA) functionalized Pt nanoparticles were connected to CNTs (adapted from [120]).

CNTs are another group of organic materials used in biosensors due to their low detection limit and good electron transfer properties. In the case of a CNT glucose biosensor, the CNTs were conjugated with glucose oxidase and was then mixed with polypyrroles (PPy). After the electropolymerization of PPy on a GCE, the biosensor showed a linear response to the glucose up to 50 mM [117]. The CNT-sulfuric acid solution was dropped on a GCE and allowed to dry. The NADH was detected through cyclic voltammograms [118]. The mixture of CNTs, chitosan, MDB, and glutamate dehydrogenase was dropped onto the surface of a GCE and left to dry before use. Thus, the resulting glutamate sensor is sensitive and stable [119]. In the complex H_2_O_2_ sensor system presented in Figure 16, activated CNTs with carboxylic groups were coated on a GCE. Dopamine functionalized Pt NPs were connected to the surface of the activated CNTs [120]. Berti synthesized a CNT thin film using the CVD method and covalently connected the CNT surface with ssDNA probes. In this specific case, the ssDNA probe was inosine-modified and guanine free. After hybridization, the guanine from the target ssDNA was easily oxidized and the oxidation signal was detected [121]. A single wall CNT-FET was reported for detecting bacterial cells. The CVD method was used to generate single wall CNT connections between catalyst islands patterned on a Si substrate. The conduction of CNT was shown to drop 50% when an *E. coli* cell stayed on the CNT [122,123]. In a biosensor for biotin-connected molecule detection, the pyrolytic graphite electrode was oxidized in order to generate carboxylic groups. Poly-L-lysine, CNT, and anti-biotin were covalently connected to the carboxylic groups. An amperometric signal was detected when biotin-connected molecules were bound to the sensor [124]. A paste electrode was fabricated using specpure carbon, CNT, Cu_2_O, and paraffin oil, which was used to differentiate amino acids with a signal to a noise ratio of 3 [125].

### 4.3. Conductive Polymers

**Figure 17.**
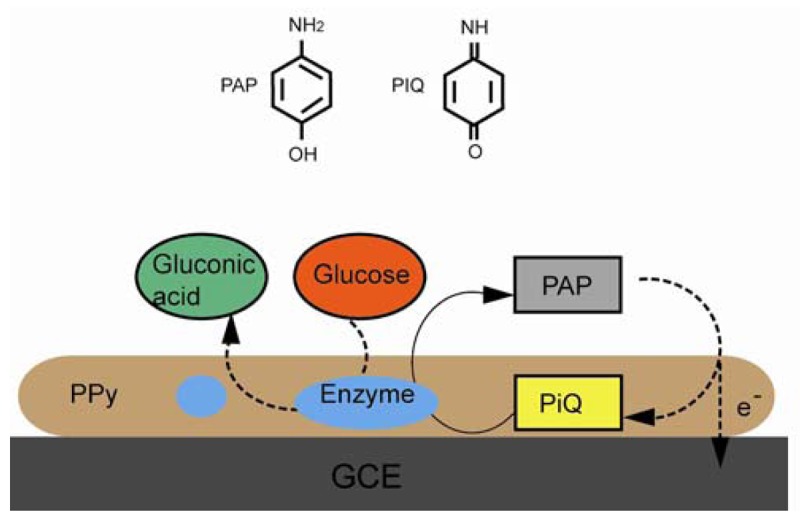
Enzyme was embedded in a PPy surface matrix coated on a GCE (adapted from [132]).

Conductive polymers are widely used in biosensors due to their sensitivity, selectivity, and the ability of integration for low-cost microfabrication [126,127]. In 1977, the high conductivity of halogen derivatives of polyacetylene was reported by Heeger, MacDiarmid, and Shirakawa [128]. The alternating singles and double bonds in conjugated polymers provide delocalized electrons, and the charge is carried by the same. The common conductive polymers include polyaniline, polythiophene, polyacetylene, and PPy [129]. The porous structure of polymers can be used as a bio-analyte immobilization matrix coupled with an electronic conduit. The most common technique for conductive polymer film fabrication is electrochemical polymerization, where the process is carried out in a monomer and bio-active species solution. In research done by Wallace, the negatively charged bio-active species were entrapped in the polymer during the electrochemical oxidation [127]. The enzyme, which serves as glucose oxidize, immobilized in the polymer matrix triggered the redox reaction of glucose. The electronic signal generated by the glucose oxidation/reduction can be relayed back to the detector through the conductive matrix. In a neuron recording application, the polymer SU-8 was used in the electrode fabrication in order to increase biocompatibility and to eliminate the tissue-electrode gap filled with a passivation layer [130]. In order to anchor the biotinylated proteins and the DNA, the NTA functionalized PPy has been coated on a Pt electrode. This reaction was proven to be as efficient as biotin-avidin reaction but avoiding the avidin layer [131]. In the example shown in Figure 17, either glucose hydrogenase or glucose oxidase was entrapped in PPy electropolymerized film on a graphite substrate. The linear detection limit of alkaline phosphate was 10^−6^ nM for glucose hydrogenase and 10^−3^ nM for glucose oxidase [132]. In another enzyme entrapment electrode, sulfite oxidase was immobilized in PPy film on a Pt electrode. The detection range for sulfite was from 0.9 to 400 mM [133]. PPy was electrogenerated on a GCE. Graphene and glucose oxidase were incorporated in the film matrix. It is shown that the enzyme-doped graphene sensor had high sensitivity towards glucose [134].

## 5. Conclusions

This review has discussed types of biosensors and applications of organic materials in biosensor systems, emphasizing SAM due to its versatility and molecular control. The interfacial properties with sensor materials of types of organic materials, such as graphene, CNTs, and electroactive polymers were discussed. Several representative applications, such as glucose sensor, pH sensor, and DNA sensor were introduced. Biosensors have attracted great attention in research due to their importance in surface science and applications. It is expected that new biosensors with novel applications will emerge in the coming years.

## Figures and Tables

**Table 1.** Summary of biosensors.

**Type**	**Sensing mechanism**	**Transducer**	**Measured property**	**References**
Resonant	The change of the viscosity, mass leads to the change of resonant frequency of the acoustic wave	Mass sensitive	Resonant frequency	[18–25]
Thermal detection	Bio-reaction results in exothermic character	Thermal	Heat of reaction or adsorption	[27]
Photometric	The change of refractive index of the solution leads to the change of refractive angle of the incident light	Optical	Surface Plasmon resonance angle	[29–32]
ISFETs	The ionic analyte diffuses into the membrane hence change the potential difference at the detecting interface	Ion-selective membrane	Surface potential	[33–40]
Electrochemical	Bio-reaction resulting in ions production or consumption will create the charge transfer across the double layer of the transducer.	Electrochemical	Potentiometric Amperometric Impedimetric	[41–43]

**Table 2.** Four types of SAM systems.

**Basic types (surface substrate)**	**Bond**	**Advantages**	**Disadvantages**	**References**
Alkanethiol and organosulfur (metal)	Metal–thiolate	Good control; most studied; gold is standard	Ligands match metal oxidation state; low stability	[67–73]
Organosilane (oxides surface; silicon dioxide, metal oxide)	S–O	Easy to handle	Multilayer defect	[64,74–76]
Alkyne and alkene (hydride-silicon surface)	Si–C	Superior stability	Oxide-free silicon is hard to obtain; multilayer defect	[77,78]
Aryl diazonium salts (carbon, metal, metal oxide, silicon surface)	Aryl–surface	Superior stability	Limited control	[79,80]

**Table 3.** Immobilization of biomolecules to biosensor systems.

**Reaction**	**Bond**	**Example**
Electrostatic	Positively (negatively) charged functional terminal and negatively (positively) charged biomolecules	Protein [84], Lipid+DNA [85]
Hydrogen	Hydrogen-electronegative atom	DNA [86], Glycan+protein [87]
Chelation	His-NTA-Cu/Ni	His-tagged Protein [88,89]
Dehydration	Amino SAM react with protein C-termini and vice versa, amide bond	Protein [90,91], Carbohydrate
Maleimide-derivated	Maleimide-thiol	Cys-protein [92]
Click	Alkyne and azide, cycloaddition	Protein [93], Carbohydrate [94,95]
Diels-Alder	Diene and dieneophile	Protein [96], Carbohydrate [97,98]
Amine-aldehyde	Secondary amine	Protein [99,100]
